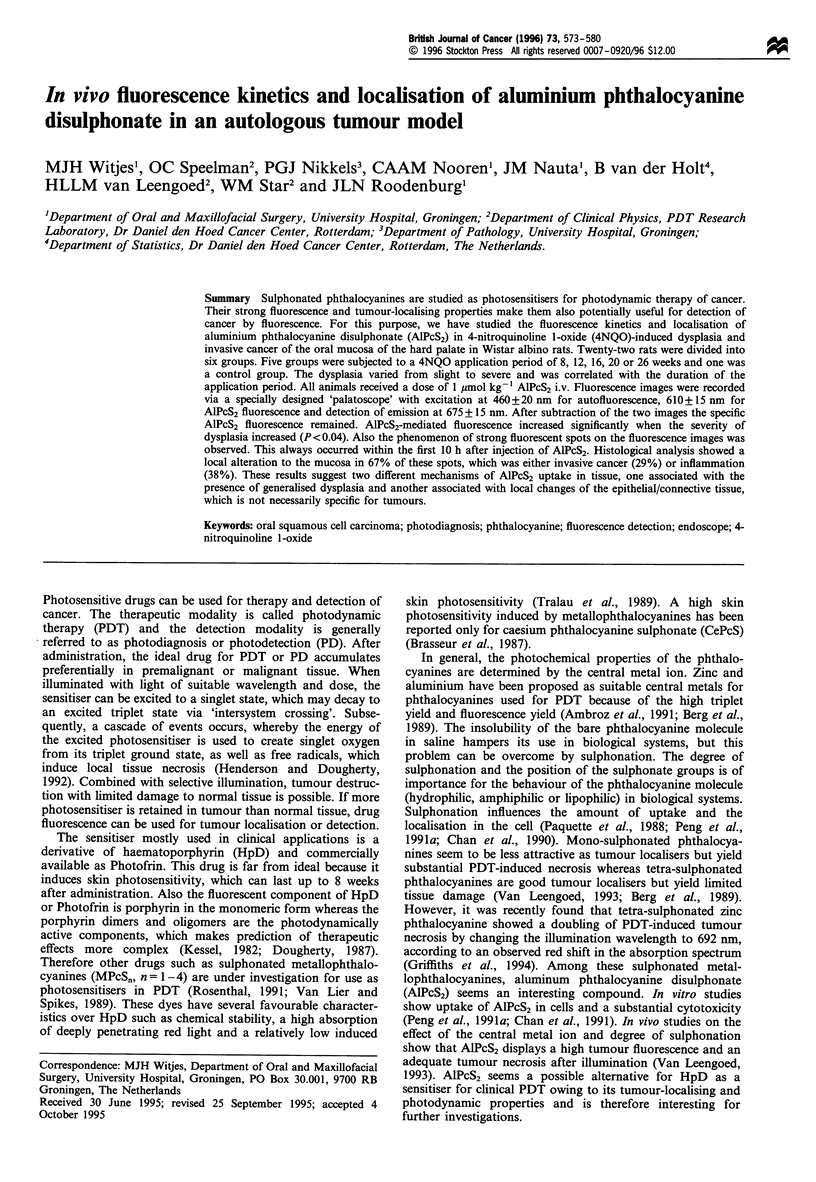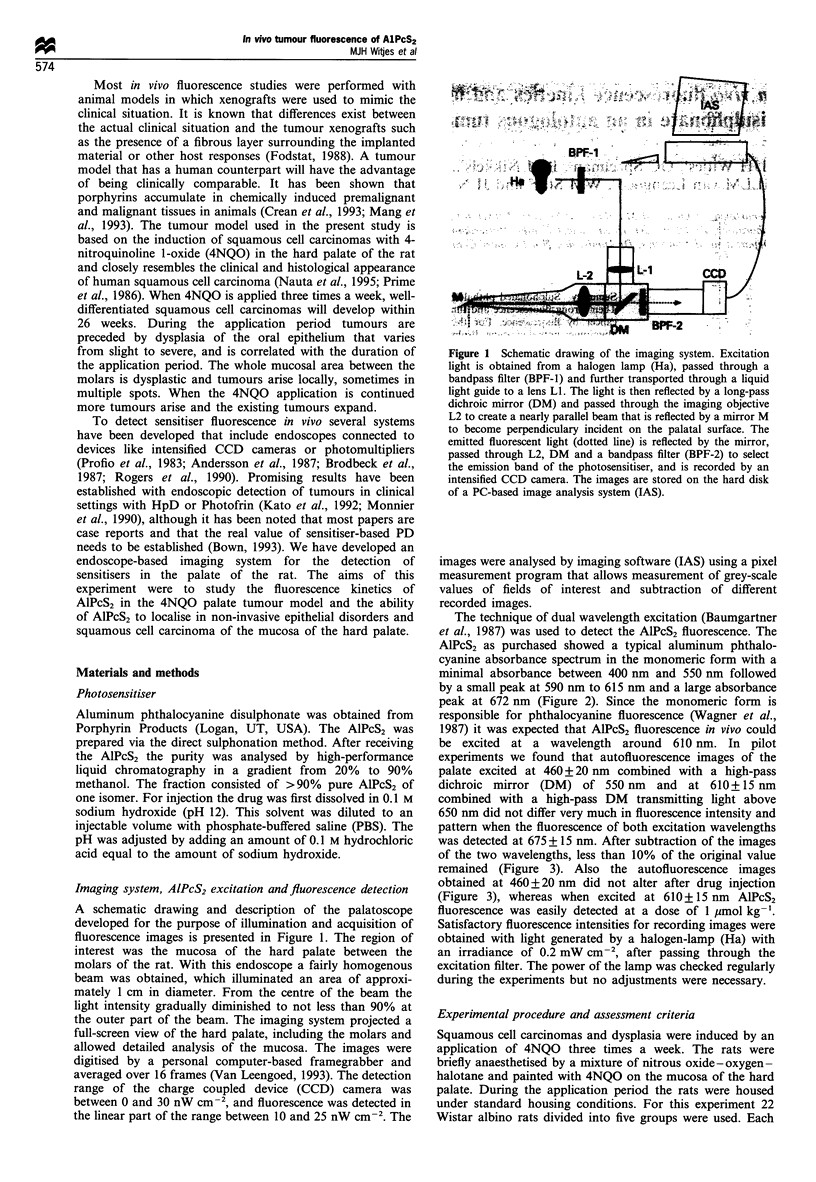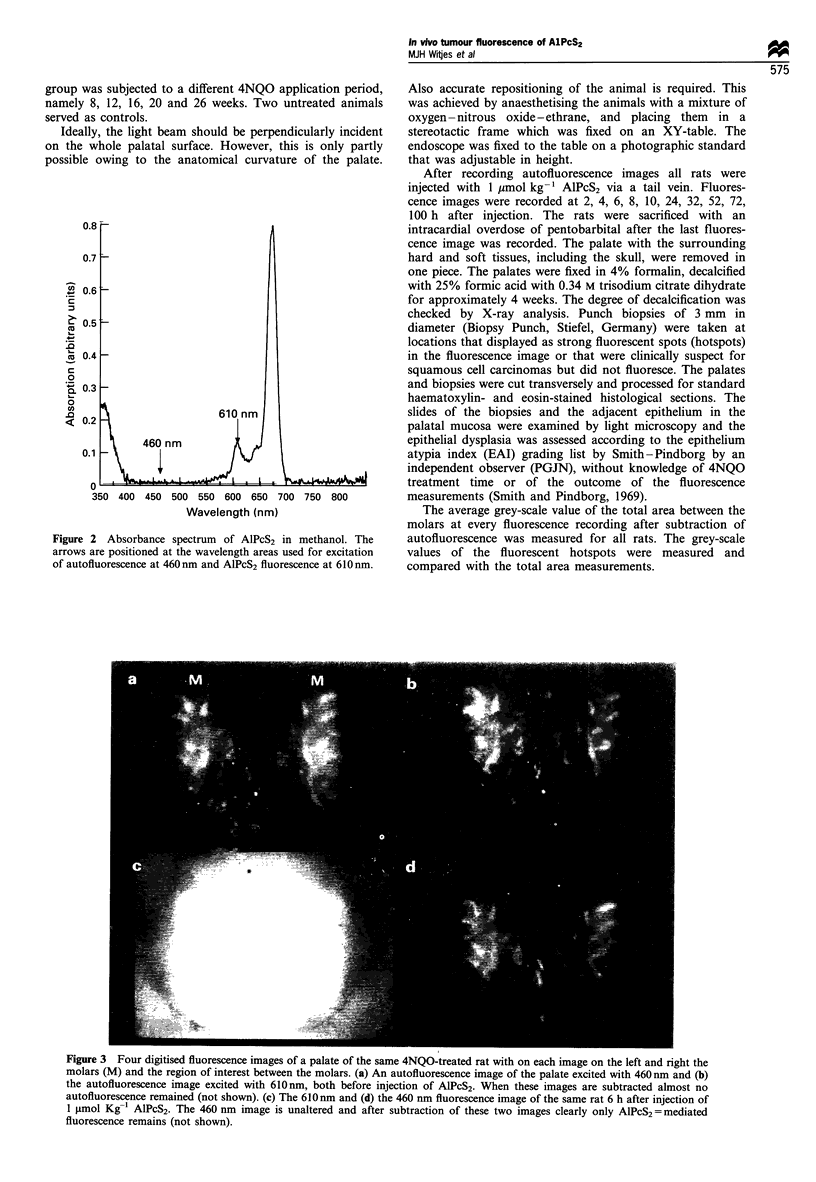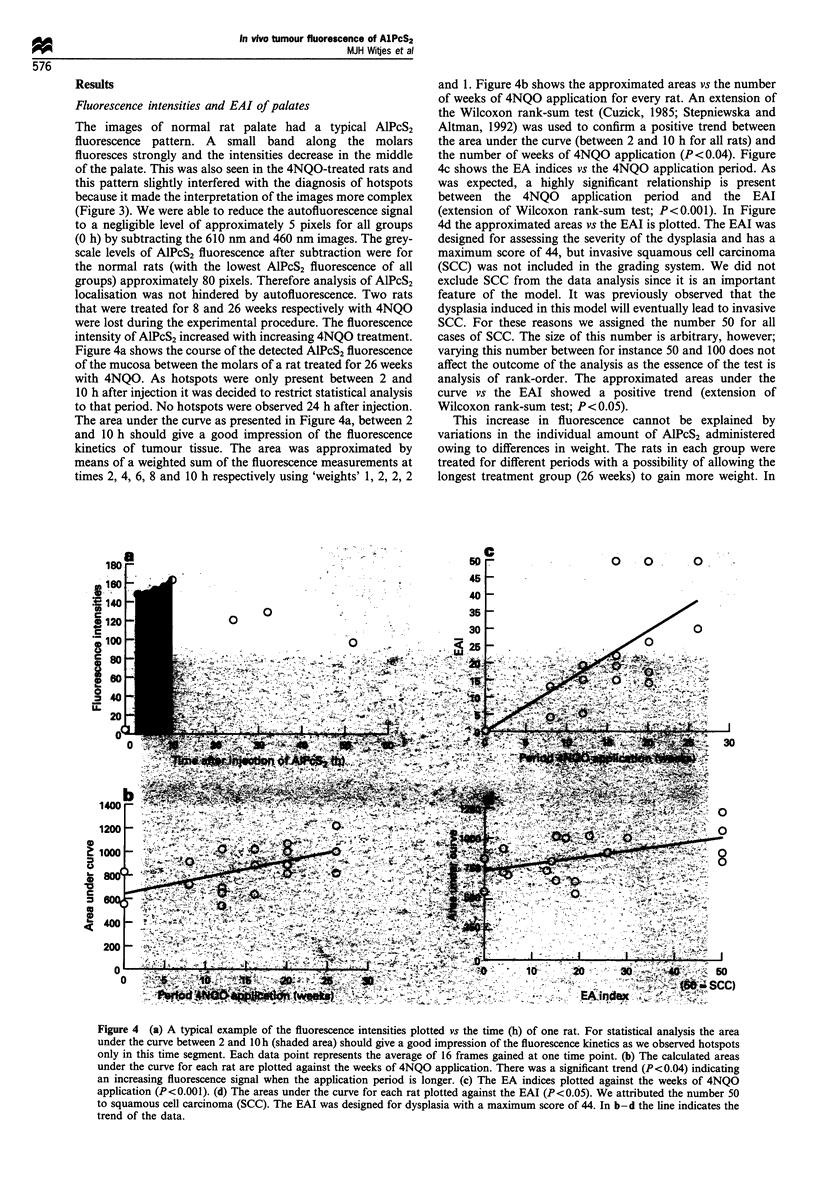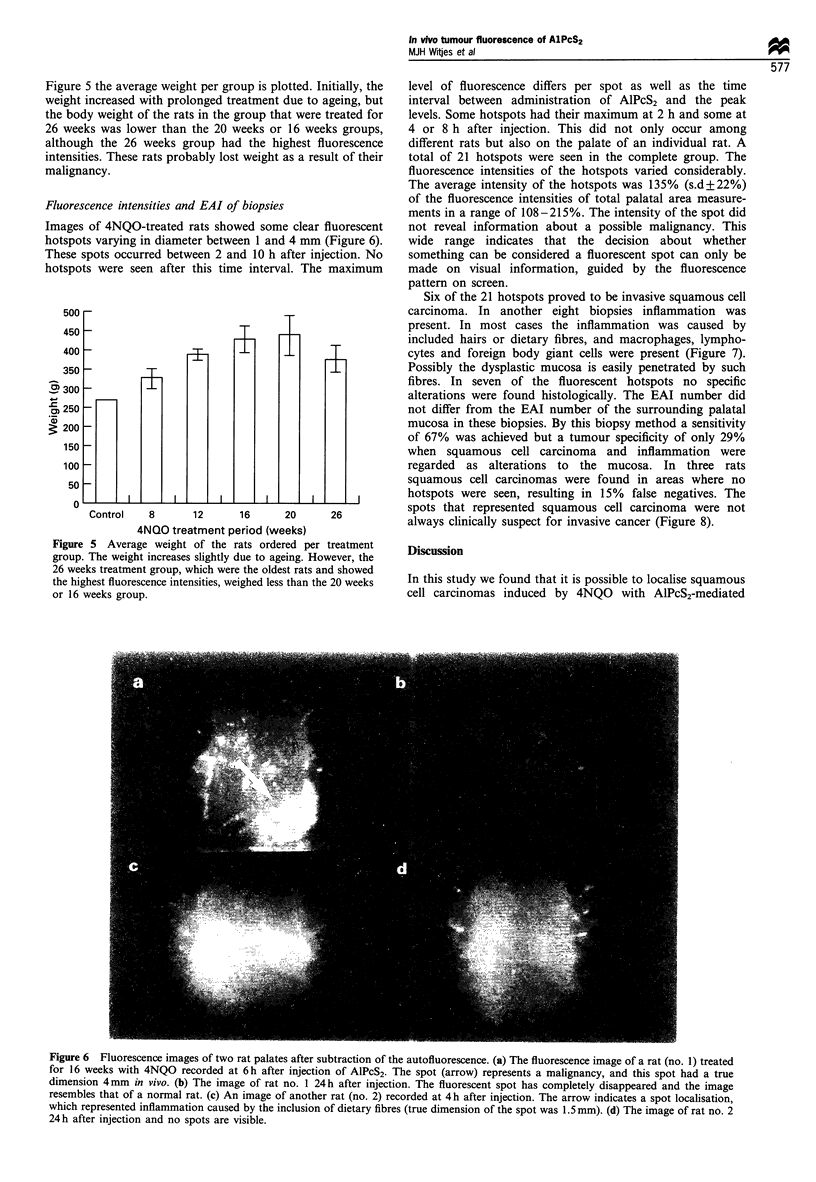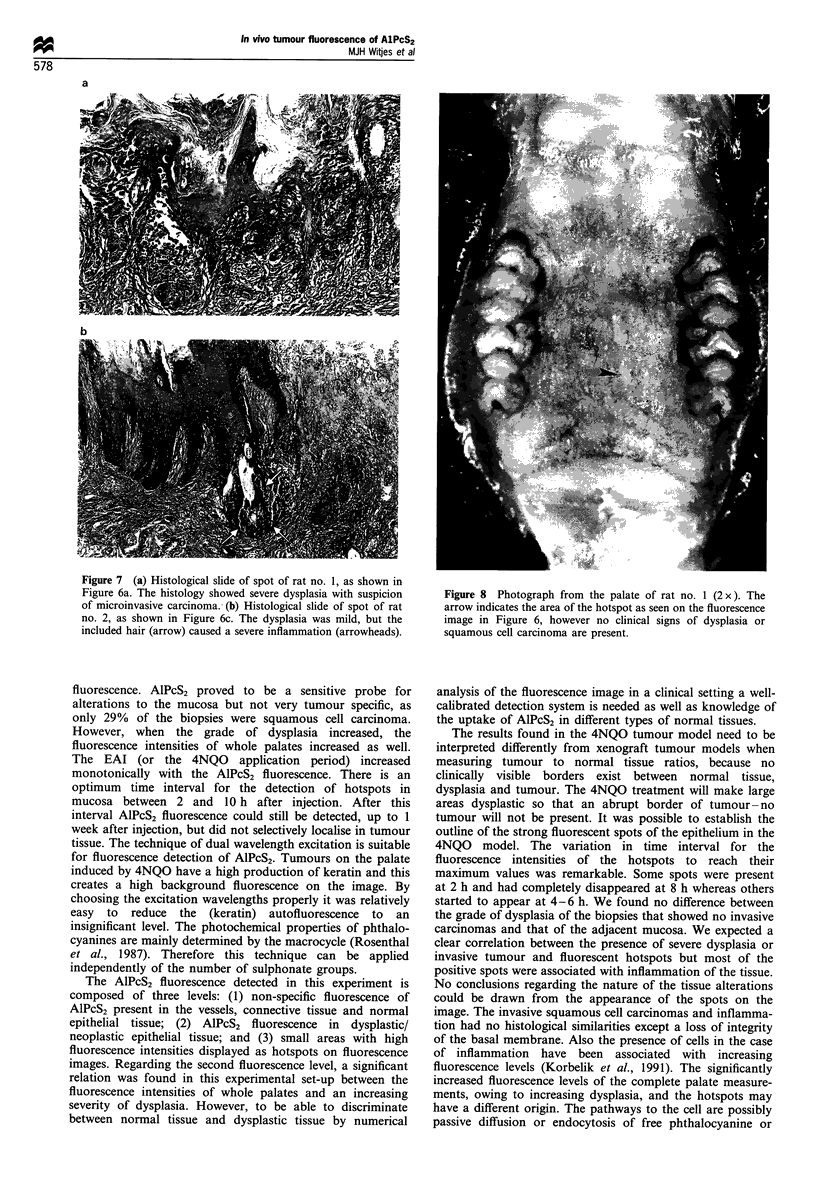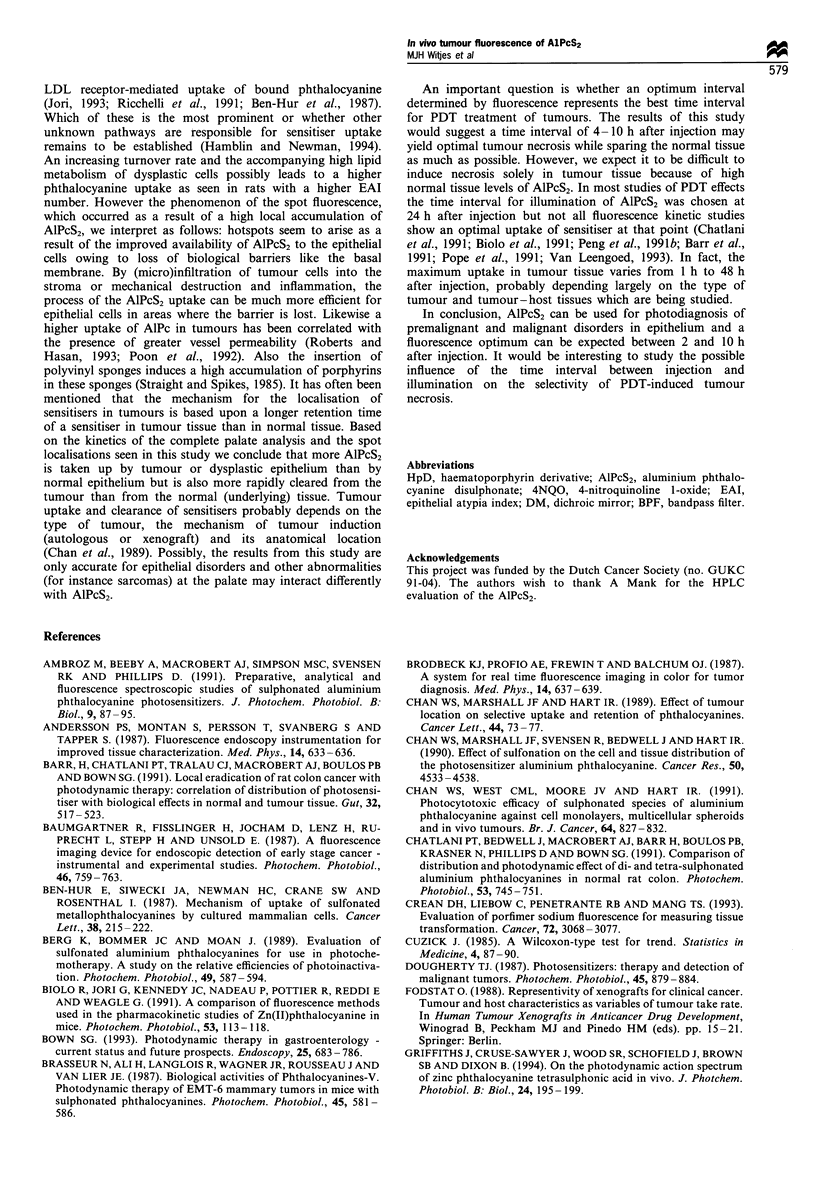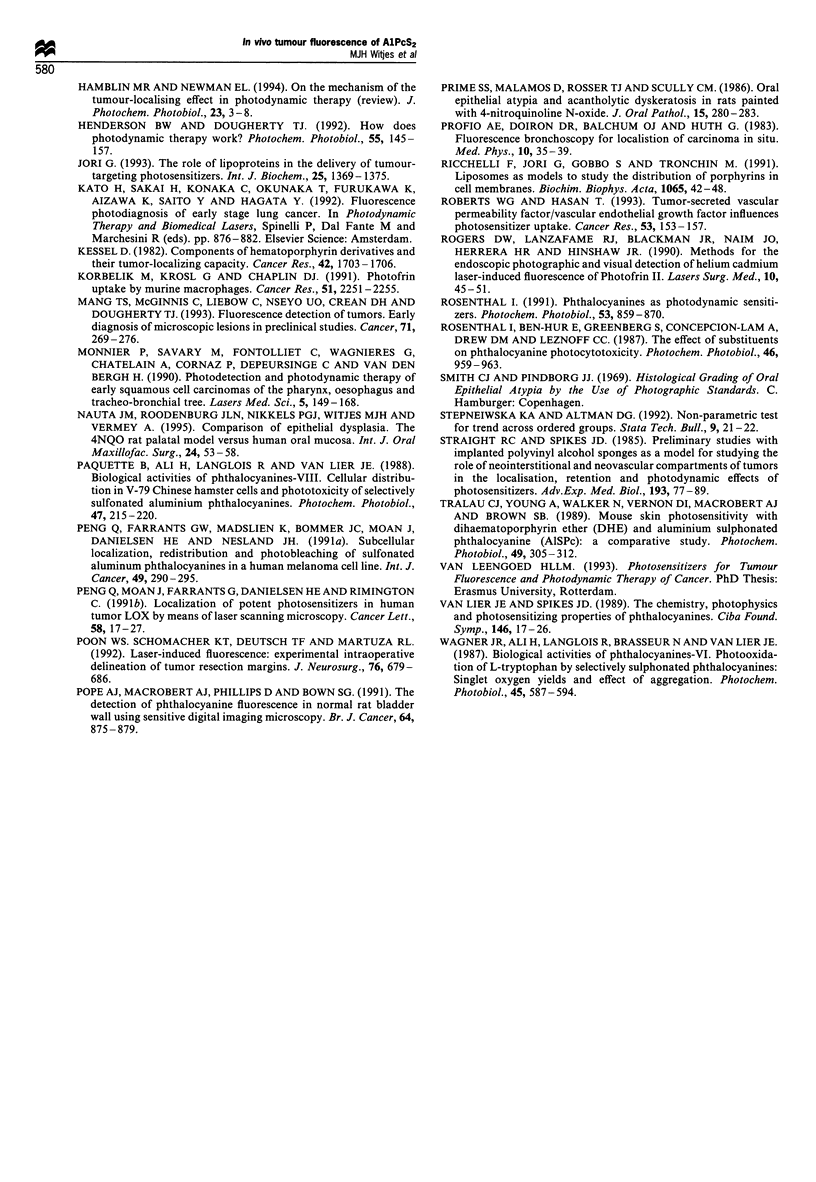# In vivo fluorescence kinetics and localisation of aluminum phthalocyanine disulphonate in an autologous tumour model.

**DOI:** 10.1038/bjc.1996.100

**Published:** 1996-03

**Authors:** M. J. Witjes, O. C. Speelman, P. G. Nikkels, C. A. Nooren, J. M. Nauta, B. van der Holt, H. L. van Leengoed, W. M. Star, J. L. Roodenburg

**Affiliations:** Department of Oral and Maxillofacial Surgery, University Hospital, Groningen, The Netherlands.

## Abstract

**Images:**


British Journal of Cancer (1996) 73, 573-580
© 1996 Stockton Press All rights reserved 0007-0920/96 $12.00

### *In vivo* fluorescence kinetics and localisation of aluminium phthalocyanine disulphonate in an autologous tumour model

MJH Witjes[1], OC Speelman[2], PGJ Nikkels[3], CAAM Nooren[1], JM Nauta[1], B van der Holt[4], HLLM van Leengoed[2], WM Star[2] and JLN Roodenburg[1]

[1]*Department of Oral and Maxillofacial Surgery, University Hospital, Groningen;* [2]*Department of Clinical Physics, PDT Research Laboratory, Dr Daniel den Hoed Cancer Center, Rotterdam;* [3]*Department of Pathology, University Hospital, Groningen;* [4]*Department of Statistics, Dr Daniel den Hoed Cancer Center, Rotterdam, The Netherlands.*

**Summary** Sulphonated phthalocyanines are studied as photosensitisers for photodynamic therapy of cancer. Their strong fluorescence and tumour-localising properties make them also potentially useful for detection of cancer by fluorescence. For this purpose, we have studied the fluorescence kinetics and localisation of aluminium phthalocyanine disulphonate ($AlPcS_2$) in 4-nitroquinoline 1-oxide (4NQO)-induced dysplasia and invasive cancer of the oral mucosa of the hard palate in Wistar albino rats. Twenty-two rats were divided into six groups. Five groups were subjected to a 4NQO application period of 8, 12, 16, 20 or 26 weeks and one was a control group. The dysplasia varied from slight to severe and was correlated with the duration of the application period. All animals received a dose of 1 $\mu$mol $kg^{-1}$ $AlPcS_2$ i.v. Fluorescence images were recorded via a specially designed 'palatoscope' with excitation at $460 \pm 20$ nm for autofluorescence, $610 \pm 15$ nm for $AlPcS_2$ fluorescence and detection of emission at $675 \pm 15$ nm. After subtraction of the two images the specific $AlPcS_2$ fluorescence remained. $AlPcS_2$-mediated fluorescence increased significantly when the severity of dysplasia increased ($P < 0.04$). Also the phenomenon of strong fluorescent spots on the fluorescence images was observed. This always occurred within the first 10 h after injection of $AlPcS_2$. Histological analysis showed a local alteration to the mucosa in 67% of these spots, which was either invasive cancer (29%) or inflammation (38%). These results suggest two different mechanisms of $AlPcS_2$ uptake in tissue, one associated with the presence of generalised dysplasia and another associated with local changes of the epithelial/connective tissue, which is not necessarily specific for tumours.

**Keywords:** oral squamous cell carcinoma; photodiagnosis; phthalocyanine; fluorescence detection; endoscope; 4-nitroquinoline 1-oxide

Photosensitive drugs can be used for therapy and detection of cancer. The therapeutic modality is called photodynamic therapy (PDT) and the detection modality is generally referred to as photodiagnosis or photodetection (PD). After administration, the ideal drug for PDT or PD accumulates preferentially in premalignant or malignant tissue. When illuminated with light of suitable wavelength and dose, the sensitiser can be excited to a singlet state, which may decay to an excited triplet state via 'intersystem crossing'. Subsequently, a cascade of events occurs, whereby the energy of the excited photosensitiser is used to create singlet oxygen from its triplet ground state, as well as free radicals, which induce local tissue necrosis (Henderson and Dougherty, 1992). Combined with selective illumination, tumour destruction with limited damage to normal tissue is possible. If more photosensitiser is retained in tumour than normal tissue, drug fluorescence can be used for tumour localisation or detection.

The sensitiser mostly used in clinical applications is a derivative of haematoporphyrin (HpD) and commercially available as Photofrin. This drug is far from ideal because it induces skin photosensitivity, which can last up to 8 weeks after administration. Also the fluorescent component of HpD or Photofrin is porphyrin in the monomeric form whereas the porphyrin dimers and oligomers are the photodynamically active components, which makes prediction of therapeutic effects more complex (Kessel, 1982; Dougherty, 1987). Therefore other drugs such as sulphonated metallophthalocyanines ($MPcS_n$, $n = 1-4$) are under investigation for use as photosensitisers in PDT (Rosenthal, 1991; Van Lier and Spikes, 1989). These dyes have several favourable characteristics over HpD such as chemical stability, a high absorption of deeply penetrating red light and a relatively low induced skin photosensitivity (Tralau *et al.*, 1989). A high skin photosensitivity induced by metallophthalocyanines has been reported only for caesium phthalocyanine sulphonate (CePcS) (Brasseur *et al.*, 1987).

In general, the photochemical properties of the phthalocyanines are determined by the central metal ion. Zinc and aluminium have been proposed as suitable central metals for phthalocyanines used for PDT because of the high triplet yield and fluorescence yield (Ambroz *et al.*, 1991; Berg *et al.*, 1989). The insolubility of the bare phthalocyanine molecule in saline hampers its use in biological systems, but this problem can be overcome by sulphonation. The degree of sulphonation and the position of the sulphonate groups is of importance for the behaviour of the phthalocyanine molecule (hydrophilic, amphiphilic or lipophilic) in biological systems. Sulphonation influences the amount of uptake and the localisation in the cell (Paquette *et al.*, 1988; Peng *et al.*, 1991*a*; Chan *et al.*, 1990). Mono-sulphonated phthalocyanines seem to be less attractive as tumour localisers but yield substantial PDT-induced necrosis whereas tetra-sulphonated phthalocyanines are good tumour localisers but yield limited tissue damage (Van Leengoed, 1993; Berg *et al.*, 1989). However, it was recently found that tetra-sulphonated zinc phthalocyanine showed a doubling of PDT-induced tumour necrosis by changing the illumination wavelength to 692 nm, according to an observed red shift in the absorption spectrum (Griffiths *et al.*, 1994). Among these sulphonated metallophthalocyanines, aluminum phthalocyanine disulphonate ($AlPcS_2$) seems an interesting compound. *In vitro* studies show uptake of $AlPcS_2$ in cells and a substantial cytotoxicity (Peng *et al.*, 1991*a*; Chan *et al.*, 1991). *In vivo* studies on the effect of the central metal ion and degree of sulphonation show that $AlPcS_2$ displays a high tumour fluorescence and an adequate tumour necrosis after illumination (Van Leengoed, 1993). $AlPcS_2$ seems a possible alternative for HpD as a sensitiser for clinical PDT owing to its tumour-localising and photodynamic properties and is therefore interesting for further investigations.

Correspondence: MJH Witjes, Department of Oral and Maxillofacial Surgery, University Hospital, Groningen, PO Box 30.001, 9700 RB Groningen, The Netherlands
Received 30 June 1995; revised 25 September 1995; accepted 4 October 1995

Most *in vivo* fluorescence studies were performed with animal models in which xenografts were used to mimic the clinical situation. It is known that differences exist between the actual clinical situation and the tumour xenografts such as the presence of a fibrous layer surrounding the implanted material or other host responses (Fodstat, 1988). A tumour model that has a human counterpart will have the advantage of being clinically comparable. It has been shown that porphyrins accumulate in chemically induced premalignant and malignant tissues in animals (Crean *et al.*, 1993; Mang *et al.*, 1993). The tumour model used in the present study is based on the induction of squamous cell carcinomas with 4-nitroquinoline 1-oxide (4NQO) in the hard palate of the rat and closely resembles the clinical and histological appearance of human squamous cell carcinoma (Nauta *et al.*, 1995; Prime *et al.*, 1986). When 4NQO is applied three times a week, well-differentiated squamous cell carcinomas will develop within 26 weeks. During the application period tumours are preceded by dysplasia of the oral epithelium that varies from slight to severe, and is correlated with the duration of the application period. The whole mucosal area between the molars is dysplastic and tumours arise locally, sometimes in multiple spots. When the 4NQO application is continued more tumours arise and the existing tumours expand.

To detect sensitiser fluorescence *in vivo* several systems have been developed that include endoscopes connected to devices like intensified CCD cameras or photomultipliers (Profio *et al.*, 1983; Andersson *et al.*, 1987; Brodbeck *et al.*, 1987; Rogers *et al.*, 1990). Promising results have been established with endoscopic detection of tumours in clinical settings with HpD or Photofrin (Kato *et al.*, 1992; Monnier *et al.*, 1990), although it has been noted that most papers are case reports and that the real value of sensitiser-based PD needs to be established (Bown, 1993). We have developed an endoscope-based imaging system for the detection of sensitisers in the palate of the rat. The aims of this experiment were to study the fluorescence kinetics of AlPcS$_2$ in the 4NQO palate tumour model and the ability of AlPcS$_2$ to localise in non-invasive epithelial disorders and squamous cell carcinoma of the mucosa of the hard palate.

### Materials and methods

### *Photosensitiser*

Aluminum phthalocyanine disulphonate was obtained from Porphyrin Products (Logan, UT, USA). The AlPcS$_2$ was prepared via the direct sulphonation method. After receiving the AlPcS$_2$ the purity was analysed by high-performance liquid chromatography in a gradient from 20% to 90% methanol. The fraction consisted of >90% pure AlPcS$_2$ of one isomer. For injection the drug was first dissolved in 0.1 M sodium hydroxide (pH 12). This solvent was diluted to an injectable volume with phosphate-buffered saline (PBS). The pH was adjusted by adding an amount of 0.1 M hydrochloric acid equal to the amount of sodium hydroxide.

### *Imaging system, AlPcS$_2$ excitation and fluorescence detection*

A schematic drawing and description of the palatoscope developed for the purpose of illumination and acquisition of fluorescence images is presented in Figure 1. The region of interest was the mucosa of the hard palate between the molars of the rat. With this endoscope a fairly homogenous beam was obtained, which illuminated an area of approximately 1 cm in diameter. From the centre of the beam the light intensity gradually diminished to not less than 90% at the outer part of the beam. The imaging system projected a full-screen view of the hard palate, including the molars and allowed detailed analysis of the mucosa. The images were digitised by a personal computer-based framegrabber and averaged over 16 frames (Van Leengoed, 1993). The detection range of the charge coupled device (CCD) camera was between 0 and 30 nW cm$^{-2}$, and fluorescence was detected in the linear part of the range between 10 and 25 nW cm$^{-2}$. The

**Figure 1** Schematic drawing of the imaging system. Excitation light is obtained from a halogen lamp (Ha), passed through a bandpass filter (BPF-1) and further transported through a liquid light guide to a lens L1. The light is then reflected by a long-pass dichroic mirror (DM) and passed through the imaging objective L2 to create a nearly parallel beam that is reflected by a mirror M to become perpendiculary incident on the palatal surface. The emitted fluorescent light (dotted line) is reflected by the mirror, passed through L2, DM and a bandpass filter (BPF-2) to select the emission band of the photosensitiser, and is recorded by an intensified CCD camera. The images are stored on the hard disk of a PC-based image analysis system (IAS).

images were analysed by imaging software (IAS) using a pixel measurement program that allows measurement of grey-scale values of fields of interest and subtraction of different recorded images.

The technique of dual wavelength excitation (Baumgartner *et al.*, 1987) was used to detect the AlPcS$_2$ fluorescence. The AlPcS$_2$ as purchased showed a typical aluminum phthalocyanine absorbance spectrum in the monomeric form with a minimal absorbance between 400 nm and 550 nm followed by a small peak at 590 nm to 615 nm and a large absorbance peak at 672 nm (Figure 2). Since the monomeric form is responsible for phthalocyanine fluorescence (Wagner *et al.*, 1987) it was expected that AlPcS$_2$ fluorescence *in vivo* could be excited at a wavelength around 610 nm. In pilot experiments we found that autofluorescence images of the palate excited at $460 \pm 20$ nm combined with a high-pass dichroic mirror (DM) of 550 nm and at $610 \pm 15$ nm combined with a high-pass DM transmitting light above 650 nm did not differ very much in fluorescence intensity and pattern when the fluorescence of both excitation wavelengths was detected at $675 \pm 15$ nm. After subtraction of the images of the two wavelengths, less than 10% of the original value remained (Figure 3). Also the autofluorescence images obtained at $460 \pm 20$ nm did not alter after drug injection (Figure 3), whereas when excited at $610 \pm 15$ nm AlPcS$_2$ fluorescence was easily detected at a dose of 1 $\mu$mol kg$^{-1}$. Satisfactory fluorescence intensities for recording images were obtained with light generated by a halogen-lamp (Ha) with an irradiance of 0.2 mW cm$^{-2}$, after passing through the excitation filter. The power of the lamp was checked regularly during the experiments but no adjustments were necessary.

### *Experimental procedure and assessment criteria*

Squamous cell carcinomas and dysplasia were induced by an application of 4NQO three times a week. The rats were briefly anaesthetised by a mixture of nitrous oxide–oxygen–halotane and painted with 4NQO on the mucosa of the hard palate. During the application period the rats were housed under standard housing conditions. For this experiment 22 Wistar albino rats divided into five groups were used. Each group was subjected to a different 4NQO application period, namely 8, 12, 16, 20 and 26 weeks. Two untreated animals served as controls.

Ideally, the light beam should be perpendicularly incident on the whole palatal surface. However, this is only partly possible owing to the anatomical curvature of the palate.

**Figure 2** Absorbance spectrum of AlPcS$_2$ in methanol. The arrows are positioned at the wavelength areas used for excitation of autofluorescence at 460 nm and AlPcS$_2$ fluorescence at 610 nm.

Also accurate repositioning of the animal is required. This was achieved by anaesthetising the animals with a mixture of oxygen–nitrous oxide–ethrane, and placing them in a stereotactic frame which was fixed on an XY-table. The endoscope was fixed to the table on a photographic standard that was adjustable in height.

After recording autofluorescence images all rats were injected with 1 $\mu$mol kg$^{-1}$ AlPcS$_2$ via a tail vein. Fluorescence images were recorded at 2, 4, 6, 8, 10, 24, 32, 52, 72, 100 h after injection. The rats were sacrificed with an intracardial overdose of pentobarbital after the last fluorescence image was recorded. The palate with the surrounding hard and soft tissues, including the skull, were removed in one piece. The palates were fixed in 4% formalin, decalcified with 25% formic acid with 0.34 M trisodium citrate dihydrate for approximately 4 weeks. The degree of decalcification was checked by X-ray analysis. Punch biopsies of 3 mm in diameter (Biopsy Punch, Stiefel, Germany) were taken at locations that displayed as strong fluorescent spots (hotspots) in the fluorescence image or that were clinically suspect for squamous cell carcinomas but did not fluoresce. The palates and biopsies were cut transversely and processed for standard haematoxylin- and eosin-stained histological sections. The slides of the biopsies and the adjacent epithelium in the palatal mucosa were examined by light microscopy and the epithelial dysplasia was assessed according to the epithelium atypia index (EAI) grading list by Smith–Pindborg by an independent observer (PGJN), without knowledge of 4NQO treatment time or of the outcome of the fluorescence measurements (Smith and Pindborg, 1969).

The average grey-scale value of the total area between the molars at every fluorescence recording after subtraction of autofluorescence was measured for all rats. The grey-scale values of the fluorescent hotspots were measured and compared with the total area measurements.

**Figure 3** Four digitised fluorescence images of a palate of the same 4NQO-treated rat with on each image on the left and right the molars (M) and the region of interest between the molars. (**a**) An autofluorescence image of the palate excited with 460 nm and (**b**) the autofluorescence image excited with 610 nm, both before injection of AlPcS$_2$. When these images are subtracted almost no autofluorescence remained (not shown). (**c**) The 610 nm and (**d**) the 460 nm fluorescence image of the same rat 6 h after injection of 1 µmol Kg$^{-1}$ AlPcS$_2$. The 460 nm image is unaltered and after subtraction of these two images clearly only AlPcS$_2$=mediated fluorescence remains (not shown).

### Results

### *Fluorescence intensities and EAI of palates*

The images of normal rat palate had a typical $AlPcS_2$ fluorescence pattern. A small band along the molars fluoresces strongly and the intensities decrease in the middle of the palate. This was also seen in the 4NQO-treated rats and this pattern slightly interfered with the diagnosis of hotspots because it made the interpretation of the images more complex (Figure 3). We were able to reduce the autofluorescence signal to a negligible level of approximately 5 pixels for all groups (0 h) by subtracting the 610 nm and 460 nm images. The grey-scale levels of $AlPcS_2$ fluorescence after subtraction were for the normal rats (with the lowest $AlPcS_2$ fluorescence of all groups) approximately 80 pixels. Therefore analysis of $AlPcS_2$ localisation was not hindered by autofluorescence. Two rats that were treated for 8 and 26 weeks respectively with 4NQO were lost during the experimental procedure. The fluorescence intensity of $AlPcS_2$ increased with increasing 4NQO treatment. Figure 4a shows the course of the detected $AlPcS_2$ fluorescence of the mucosa between the molars of a rat treated for 26 weeks with 4NQO. As hotspots were only present between 2 and 10 h after injection it was decided to restrict statistical analysis to that period. No hotspots were observed 24 h after injection. The area under the curve as presented in Figure 4a, between 2 and 10 h should give a good impression of the fluorescence kinetics of tumour tissue. The area was approximated by means of a weighted sum of the fluorescence measurements at times 2, 4, 6, 8 and 10 h respectively using 'weights' 1, 2, 2, 2 and 1. Figure 4b shows the approximated areas *vs* the number of weeks of 4NQO application for every rat. An extension of the Wilcoxon rank-sum test (Cuzick, 1985; Stepniewska and Altman, 1992) was used to confirm a positive trend between the area under the curve (between 2 and 10 h for all rats) and the number of weeks of 4NQO application ($P<0.04$). Figure 4c shows the EA indices *vs* the 4NQO application period. As was expected, a highly significant relationship is present between the 4NQO application period and the EAI (extension of Wilcoxon rank-sum test; $P<0.001$). In Figure 4d the approximated areas *vs* the EAI is plotted. The EAI was designed for assessing the severity of the dysplasia and has a maximum score of 44, but invasive squamous cell carcinoma (SCC) was not included in the grading system. We did not exclude SCC from the data analysis since it is an important feature of the model. It was previously observed that the dysplasia induced in this model will eventually lead to invasive SCC. For these reasons we assigned the number 50 for all cases of SCC. The size of this number is arbitrary, however; varying this number between for instance 50 and 100 does not affect the outcome of the analysis as the essence of the test is analysis of rank-order. The approximated areas under the curve *vs* the EAI showed a positive trend (extension of Wilcoxon rank-sum test; $P<0.05$).

This increase in fluorescence cannot be explained by variations in the individual amount of $AlPcS_2$ administered owing to differences in weight. The rats in each group were treated for different periods with a possibility of allowing the longest treatment group (26 weeks) to gain more weight. In

**Figure 4** (**a**) A typical example of the fluorescence intensities plotted *vs* the time (h) of one rat. For statistical analysis the area under the curve between 2 and 10 h (shaded area) should give a good impression of the fluorescence kinetics as we observed hotspots only in this time segment. Each data point represents the average of 16 frames gained at one time point. (**b**) The calculated areas under the curve for each rat are plotted against the weeks of 4NQO application. There was a significant trend ($P<0.04$) indicating an increasing fluorescence signal when the application period is longer. (**c**) The EA indices plotted against the weeks of 4NQO application ($P<0.001$). (**d**) The areas under the curve for each rat plotted against the EAI ($P<0.05$). We attributed the number 50 to squamous cell carcinoma (SCC). The EAI was designed for dysplasia with a maximum score of 44. In **b**–**d** the line indicates the trend of the data.

Figure 5 the average weight per group is plotted. Initially, the weight increased with prolonged treatment due to ageing, but the body weight of the rats in the group that were treated for 26 weeks was lower than the 20 weeks or 16 weeks groups, although the 26 weeks group had the highest fluorescence intensities. These rats probably lost weight as a result of their malignancy.

*Fluorescence intensities and EAI of biopsies*

Images of 4NQO-treated rats showed some clear fluorescent hotspots varying in diameter between 1 and 4 mm (Figure 6). These spots occurred between 2 and 10 h after injection. No hotspots were seen after this time interval. The maximum level of fluorescence differs per spot as well as the time interval between administration of $AlPcS_2$ and the peak levels. Some hotspots had their maximum at 2 h and some at 4 or 8 h after injection. This did not only occur among different rats but also on the palate of an individual rat. A total of 21 hotspots were seen in the complete group. The fluorescence intensities of the hotspots varied considerably. The average intensity of the hotspots was 135% (s.d $\pm$22%) of the fluorescence intensities of total palatal area measurements in a range of 108–215%. The intensity of the spot did not reveal information about a possible malignancy. This wide range indicates that the decision about whether something can be considered a fluorescent spot can only be made on visual information, guided by the fluorescence pattern on screen.

**Figure 5** Average weight of the rats ordered per treatment group. The weight increases slightly due to ageing. However, the 26 weeks treatment group, which were the oldest rats and showed the highest fluorescence intensities, weighed less than the 20 weeks or 16 weeks group.

Six of the 21 hotspots proved to be invasive squamous cell carcinoma. In another eight biopsies inflammation was present. In most cases the inflammation was caused by included hairs or dietary fibres, and macrophages, lymphocytes and foreign body giant cells were present (Figure 7). Possibly the dysplastic mucosa is easily penetrated by such fibres. In seven of the fluorescent hotspots no specific alterations were found histologically. The EAI number did not differ from the EAI number of the surrounding palatal mucosa in these biopsies. By this biopsy method a sensitivity of 67% was achieved but a tumour specificity of only 29% when squamous cell carcinoma and inflammation were regarded as alterations to the mucosa. In three rats squamous cell carcinomas were found in areas where no hotspots were seen, resulting in 15% false negatives. The spots that represented squamous cell carcinoma were not always clinically suspect for invasive cancer (Figure 8).

### Discussion

In this study we found that it is possible to localise squamous cell carcinomas induced by 4NQO with $AlPcS_2$-mediated

**Figure 6** Fluorescence images of two rat palates after subtraction of the autofluorescence. (**a**) The fluorescence image of a rat (no. 1) treated for 16 weeks with 4NQO recorded at 6 h after injection of $AlPcS_2$. The spot (arrow) represents a malignancy, and this spot had a true dimension 4 mm *in vivo*. (**b**) The image of rat no. 1 24 h after injection. The fluorescent spot has completely disappeared and the image resembles that of a normal rat. (**c**) An image of another rat (no. 2) recorded at 4 h after injection. The arrow indicates a spot localisation, which represented inflammation caused by the inclusion of dietary fibres (true dimension of the spot was 1.5 mm). (**d**) The image of rat no. 2 24 h after injection and no spots are visible.

a
b

**Figure 7** (**a**) Histological slide of spot of rat no. 1, as shown in Figure 6a. The histology showed severe dysplasia with suspicion of microinvasive carcinoma. (**b**) Histological slide of spot of rat no. 2, as shown in Figure 6c. The dysplasia was mild, but the included hair (arrow) caused a severe inflammation (arrowheads).

**Figure 8** Photograph from the palate of rat no. 1 (2 ×). The arrow indicates the area of the hotspot as seen on the fluorescence image in Figure 6, however no clinical signs of dysplasia or squamous cell carcinoma are present.

fluorescence. $AlPcS_2$ proved to be a sensitive probe for alterations to the mucosa but not very tumour specific, as only 29% of the biopsies were squamous cell carcinoma. However, when the grade of dysplasia increased, the fluorescence intensities of whole palates increased as well. The EAI (or the 4NQO application period) increased monotonically with the $AlPcS_2$ fluorescence. There is an optimum time interval for the detection of hotspots in mucosa between 2 and 10 h after injection. After this interval $AlPcS_2$ fluorescence could still be detected, up to 1 week after injection, but did not selectively localise in tumour tissue. The technique of dual wavelength excitation is suitable for fluorescence detection of $AlPcS_2$. Tumours on the palate induced by 4NQO have a high production of keratin and this creates a high background fluorescence on the image. By choosing the excitation wavelengths properly it was relatively easy to reduce the (keratin) autofluorescence to an insignificant level. The photochemical properties of phthalocyanines are mainly determined by the macrocycle (Rosenthal *et al.*, 1987). Therefore this technique can be applied independently of the number of sulphonate groups.

The $AlPcS_2$ fluorescence detected in this experiment is composed of three levels: (1) non-specific fluorescence of $AlPcS_2$ present in the vessels, connective tissue and normal epithelial tissue; (2) $AlPcS_2$ fluorescence in dysplastic/neoplastic epithelial tissue; and (3) small areas with high fluorescence intensities displayed as hotspots on fluorescence images. Regarding the second fluorescence level, a significant relation was found in this experimental set-up between the fluorescence intensities of whole palates and an increasing severity of dysplasia. However, to be able to discriminate between normal tissue and dysplastic tissue by numerical analysis of the fluorescence image in a clinical setting a well-calibrated detection system is needed as well as knowledge of the uptake of $AlPcS_2$ in different types of normal tissues.

The results found in the 4NQO tumour model need to be interpreted differently from xenograft tumour models when measuring tumour to normal tissue ratios, because no clinically visible borders exist between normal tissue, dysplasia and tumour. The 4NQO treatment will make large areas dysplastic so that an abrupt border of tumour–no tumour will not be present. It was possible to establish the outline of the strong fluorescent spots of the epithelium in the 4NQO model. The variation in time interval for the fluorescence intensities of the hotspots to reach their maximum values was remarkable. Some spots were present at 2 h and had completely disappeared at 8 h whereas others started to appear at 4–6 h. We found no difference between the grade of dysplasia of the biopsies that showed no invasive carcinomas and that of the adjacent mucosa. We expected a clear correlation between the presence of severe dysplasia or invasive tumour and fluorescent hotspots but most of the positive spots were associated with inflammation of the tissue. No conclusions regarding the nature of the tissue alterations could be drawn from the appearance of the spots on the image. The invasive squamous cell carcinomas and inflammation had no histological similarities except a loss of integrity of the basal membrane. Also the presence of cells in the case of inflammation have been associated with increasing fluorescence levels (Korbelik *et al.*, 1991). The significantly increased fluorescence levels of the complete palate measurements, owing to increasing dysplasia, and the hotspots may have a different origin. The pathways to the cell are possibly passive diffusion or endocytosis of free phthalocyanine or

LDL receptor-mediated uptake of bound phthalocyanine (Jori, 1993; Ricchelli *et al.*, 1991; Ben-Hur *et al.*, 1987). Which of these is the most prominent or whether other unknown pathways are responsible for sensitiser uptake remains to be established (Hamblin and Newman, 1994). An increasing turnover rate and the accompanying high lipid metabolism of dysplastic cells possibly leads to a higher phthalocyanine uptake as seen in rats with a higher EAI number. However the phenomenon of the spot fluorescence, which occurred as a result of a high local accumulation of $AlPcS_2$, we interpret as follows: hotspots seem to arise as a result of the improved availability of $AlPcS_2$ to the epithelial cells owing to loss of biological barriers like the basal membrane. By (micro)infiltration of tumour cells into the stroma or mechanical destruction and inflammation, the process of the $AlPcS_2$ uptake can be much more efficient for epithelial cells in areas where the barrier is lost. Likewise a higher uptake of AlPc in tumours has been correlated with the presence of greater vessel permeability (Roberts and Hasan, 1993; Poon *et al.*, 1992). Also the insertion of polyvinyl sponges induces a high accumulation of porphyrins in these sponges (Straight and Spikes, 1985). It has often been mentioned that the mechanism for the localisation of sensitisers in tumours is based upon a longer retention time of a sensitiser in tumour tissue than in normal tissue. Based on the kinetics of the complete palate analysis and the spot localisations seen in this study we conclude that more $AlPcS_2$ is taken up by tumour or dysplastic epithelium than by normal epithelium but is also more rapidly cleared from the tumour than from the normal (underlying) tissue. Tumour uptake and clearance of sensitisers probably depends on the type of tumour, the mechanism of tumour induction (autologous or xenograft) and its anatomical location (Chan *et al.*, 1989). Possibly, the results from this study are only accurate for epithelial disorders and other abnormalities (for instance sarcomas) at the palate may interact differently with $AlPcS_2$.

An important question is whether an optimum interval determined by fluorescence represents the best time interval for PDT treatment of tumours. The results of this study would suggest a time interval of 4–10 h after injection may yield optimal tumour necrosis while sparing the normal tissue as much as possible. However, we expect it to be difficult to induce necrosis solely in tumour tissue because of high normal tissue levels of $AlPcS_2$. In most studies of PDT effects the time interval for illumination of $AlPcS_2$ was chosen at 24 h after injection but not all fluorescence kinetic studies show an optimal uptake of sensitiser at that point (Chatlani *et al.*, 1991; Biolo *et al.*, 1991; Peng *et al.*, 1991*b*; Barr *et al.*, 1991; Pope *et al.*, 1991; Van Leengoed, 1993). In fact, the maximum uptake in tumour tissue varies from 1 h to 48 h after injection, probably depending largely on the type of tumour and tumour–host tissues which are being studied.

In conclusion, $AlPcS_2$ can be used for photodiagnosis of premalignant and malignant disorders in epithelium and a fluorescence optimum can be expected between 2 and 10 h after injection. It would be interesting to study the possible influence of the time interval between injection and illumination on the selectivity of PDT-induced tumour necrosis.

### Abbreviations

HpD, haematoporphyrin derivative; $AlPcS_2$, aluminium phthalocyanine disulphonate; 4NQO, 4-nitroquinoline 1-oxide; EAI, epithelial atypia index; DM, dichroic mirror; BPF, bandpass filter.

### Acknowledgements

This project was funded by the Dutch Cancer Society (no. GUKC 91-04). The authors wish to thank A Mank for the HPLC evaluation of the $AlPcS_2$.

### References

AMBROZ M, BEEBY A, MACROBERT AJ, SIMPSON MSC, SVENSEN RK AND PHILLIPS D. (1991). Preparative, analytical and fluorescence spectroscopic studies of sulphonated aluminium phthalocyanine photosensitizers. *J. Photochem. Photobiol. B: Biol.*, **9**, 87–95.

ANDERSSON PS, MONTAN S, PERSSON T, SVANBERG S AND TAPPER S. (1987). Fluorescence endoscopy instrumentation for improved tissue characterization. *Med. Phys.*, **14**, 633–636.

BARR, H, CHATLANI PT, TRALAU CJ, MACROBERT AJ, BOULOS PB AND BOWN SG. (1991). Local eradication of rat colon cancer with photodynamic therapy: correlation of distribution of photosensitiser with biological effects in normal and tumour tissue. *Gut*, **32**, 517–523.

BAUMGARTNER R, FISSLINGER H, JOCHAM D, LENZ H, RUPRECHT L, STEPP H AND UNSOLD E. (1987). A fluorescence imaging device for endoscopic detection of early stage cancer - instrumental and experimental studies. *Photochem. Photobiol.*, **46**, 759–763.

BEN-HUR E, SIWECKI JA, NEWMAN HC, CRANE SW AND ROSENTHAL I. (1987). Mechanism of uptake of sulfonated metallophthalocyanines by cultured mammalian cells. *Cancer Lett.*, **38**, 215–222.

BERG K, BOMMER JC AND MOAN J. (1989). Evaluation of sulfonated aluminium phthalocyanines for use in photochemotherapy. A study on the relative efficiencies of photoinactivation. *Photochem. Photobiol.*, **49**, 587–594.

BIOLO R, JORI G, KENNEDY JC, NADEAU P, POTTIER R, REDDI E AND WEAGLE G. (1991). A comparison of fluorescence methods used in the pharmacokinetic studies of Zn(II)phthalocyanine in mice. *Photochem. Photobiol.*, **53**, 113–118.

BOWN SG. (1993). Photodynamic therapy in gastroenterology - current status and future prospects. *Endoscopy*, **25**, 683–786.

BRASSEUR N, ALI H, LANGLOIS R, WAGNER JR, ROUSSEAU J AND VAN LIER JE. (1987). Biological activities of Phthalocyanines-V. Photodynamic therapy of EMT-6 mammary tumors in mice with sulphonated phthalocyanines. *Photochem. Photobiol.*, **45**, 581–586.

BRODBECK KJ, PROFIO AE, FREWIN T AND BALCHUM OJ. (1987). A system for real time fluorescence imaging in color for tumor diagnosis. *Med. Phys.*, **14**, 637–639.

CHAN WS, MARSHALL JF AND HART IR. (1989). Effect of tumour location on selective uptake and retention of phthalocyanines. *Cancer Lett.*, **44**, 73–77.

CHAN WS, MARSHALL JF, SVENSEN R, BEDWELL J AND HART IR. (1990). Effect of sulfonation on the cell and tissue distribution of the photosensitizer aluminium phthalocyanine. *Cancer Res.*, **50**, 4533–4538.

CHAN WS, WEST CML, MOORE JV AND HART IR. (1991). Photocytotoxic efficacy of sulphonated species of aluminium phthalocyanine against cell monolayers, multicellular spheroids and in vivo tumours. *Br. J. Cancer*, **64**, 827–832.

CHATLANI PT, BEDWELL J, MACROBERT AJ, BARR H, BOULOS PB, KRASNER N, PHILLIPS D AND BOWN SG. (1991). Comparison of distribution and photodynamic effect of di- and tetra-sulphonated aluminium phthalocyanines in normal rat colon. *Photochem. Photobiol.*, **53**, 745–751.

CREAN DH, LIEBOW C, PENETRANTE RB AND MANG TS. (1993). Evaluation of porfimer sodium fluorescence for measuring tissue transformation. *Cancer*, **72**, 3068–3077.

CUZICK J. (1985). A Wilcoxon-type test for trend. *Statistics in Medicine*, **4**, 87–90.

DOUGHERTY TJ. (1987). Photosensitizers: therapy and detection of malignant tumors. *Photochem. Photobiol.*, **45**, 879–884.

FODSTAT O. (1988). Representivity of xenografts for clinical cancer. Tumour and host characteristics as variables of tumour take rate. In *Human Tumour Xenografts in Anticancer Drug Development*, Winograd B, Peckham MJ and Pinedo HM (eds). pp. 15–21. Springer: Berlin.

GRIFFITHS J, CRUSE-SAWYER J, WOOD SR, SCHOFIELD J, BROWN SB AND DIXON B. (1994). On the photodynamic action spectrum of zinc phthalocyanine tetrasulphonic acid in vivo. *J. Photchem. Photobiol. B: Biol.*, **24**, 195–199.

HAMBLIN MR AND NEWMAN EL. (1994). On the mechanism of the tumour-localising effect in photodynamic therapy (review). *J. Photochem. Photobiol.*, **23**, 3–8.

HENDERSON BW AND DOUGHERTY TJ. (1992). How does photodynamic therapy work? *Photochem. Photobiol.*, **55**, 145–157.

JORI G. (1993). The role of lipoproteins in the delivery of tumour-targeting photosensitizers. *Int. J. Biochem.*, **25**, 1369–1375.

KATO H, SAKAI H, KONAKA C, OKUNAKA T, FURUKAWA K, AIZAWA K, SAITO Y AND HAGATA Y. (1992). Fluorescence photodiagnosis of early stage lung cancer. In *Photodynamic Therapy and Biomedical Lasers*, Spinelli P, Dal Fante M and Marchesini R (eds). pp. 876–882. Elsevier Science: Amsterdam.

KESSEL D. (1982). Components of hematoporphyrin derivatives and their tumor-localizing capacity. *Cancer Res.*, **42**, 1703–1706.

KORBELIK M, KROSL G AND CHAPLIN DJ. (1991). Photofrin uptake by murine macrophages. *Cancer Res.*, **51**, 2251–2255.

MANG TS, McGINNIS C, LIEBOW C, NSEYO UO, CREAN DH AND DOUGHERTY TJ. (1993). Fluorescence detection of tumors. Early diagnosis of microscopic lesions in preclinical studies. *Cancer*, **71**, 269–276.

MONNIER P, SAVARY M, FONTOLLIET C, WAGNIERES G, CHATELAIN A, CORNAZ P, DEPEURSINGE C AND VAN DEN BERGH H. (1990). Photodetection and photodynamic therapy of early squamous cell carcinomas of the pharynx, oesophagus and tracheo-bronchial tree. *Lasers Med. Sci.*, **5**, 149–168.

NAUTA JM, ROODENBURG JLN, NIKKELS PGJ, WITJES MJH AND VERMEY A. (1995). Comparison of epithelial dysplasia. The 4NQO rat palatal model versus human oral mucosa. *Int. J. Oral Maxillofac. Surg.*, **24**, 53–58.

PAQUETTE B, ALI H, LANGLOIS R AND VAN LIER JE. (1988). Biological activities of phthalocyanines-VIII. Cellular distribution in V-79 Chinese hamster cells and phototoxicity of selectively sulfonated aluminium phthalocyanines. *Photochem. Photobiol.*, **47**, 215–220.

PENG Q, FARRANTS GW, MADSLIEN K, BOMMER JC, MOAN J, DANIELSEN HE AND NESLAND JH. (1991*a*). Subcellular localization, redistribution and photobleaching of sulfonated aluminum phthalocyanines in a human melanoma cell line. *Int. J. Cancer*, **49**, 290–295.

PENG Q, MOAN J, FARRANTS G, DANIELSEN HE AND RIMINGTON C. (1991*b*). Localization of potent photosensitizers in human tumor LOX by means of laser scanning microscopy. *Cancer Lett.*, **58**, 17–27.

POON WS. SCHOMACHER KT, DEUTSCH TF AND MARTUZA RL. (1992). Laser-induced fluorescence: experimental intraoperative delineation of tumor resection margins. *J. Neurosurg.*, **76**, 679–686.

POPE AJ, MACROBERT AJ, PHILLIPS D AND BOWN SG. (1991). The detection of phthalocyanine fluorescence in normal rat bladder wall using sensitive digital imaging microscopy. *Br. J. Cancer*, **64**, 875–879.

PRIME SS, MALAMOS D, ROSSER TJ AND SCULLY CM. (1986). Oral epithelial atypia and acantholytic dyskeratosis in rats painted with 4-nitroquinoline N-oxide. *J. Oral Pathol.*, **15**, 280–283.

PROFIO AE, DOIRON DR, BALCHUM OJ AND HUTH G. (1983). Fluorescence bronchoscopy for localistion of carcinoma in situ. *Med. Phys.*, **10**, 35–39.

RICCHELLI F, JORI G, GOBBO S AND TRONCHIN M. (1991). Liposomes as models to study the distribution of porphyrins in cell membranes. *Biochim. Biophys. Acta*, **1065**, 42–48.

ROBERTS WG AND HASAN T. (1993). Tumor-secreted vascular permeability factor/vascular endothelial growth factor influences photosensitizer uptake. *Cancer Res.*, **53**, 153–157.

ROGERS DW, LANZAFAME RJ, BLACKMAN JR, NAIM JO, HERRERA HR AND HINSHAW JR. (1990). Methods for the endoscopic photographic and visual detection of helium cadmium laser-induced fluorescence of Photofrin II. *Lasers Surg. Med.*, **10**, 45–51.

ROSENTHAL I. (1991). Phthalocyanines as photodynamic sensitizers. *Photochem. Photobiol.*, **53**, 859–870.

ROSENTHAL I, BEN-HUR E, GREENBERG S, CONCEPCION-LAM A, DREW DM AND LEZNOFF CC. (1987). The effect of substituents on phthalocyanine photocytotoxicity. *Photochem. Photobiol.*, **46**, 959–963.

SMITH CJ AND PINDBORG JJ. (1969). *Histological Grading of Oral Epithelial Atypia by the Use of Photographic Standards*. C. Hamburger: Copenhagen.

STEPNEIWSKA KA AND ALTMAN DG. (1992). Non-parametric test for trend across ordered groups. *Stata Tech. Bull.*, **9**, 21–22.

STRAIGHT RC AND SPIKES JD. (1985). Preliminary studies with implanted polyvinyl alcohol sponges as a model for studying the role of neointerstitional and neovascular compartments of tumors in the localisation, retention and photodynamic effects of photosensitizers. *Adv.Exp. Med. Biol.*, **193**, 77–89.

TRALAU CJ, YOUNG A, WALKER N, VERNON DI, MACROBERT AJ AND BROWN SB. (1989). Mouse skin photosensitivity with dihaematoporphyrin ether (DHE) and aluminium sulphonated phthalocyanine (AlSPc): a comparative study. *Photochem. Photobiol.*, **49**, 305–312.

VAN LEENGOED HLLM. (1993). *Photosensitizers for Tumour Fluorescence and Photodynamic Therapy of Cancer*. PhD Thesis: Erasmus University, Rotterdam.

VAN LIER JE AND SPIKES JD. (1989). The chemistry, photophysics and photosensitizing properties of phthalocyanines. *Ciba Found. Symp.*, **146**, 17–26.

WAGNER JR, ALI H, LANGLOIS R, BRASSEUR N AND VAN LIER JE. (1987). Biological activities of phthalocyanines-VI. Photooxidation of L-tryptophan by selectively sulphonated phthalocyanines: Singlet oxygen yields and effect of aggregation. *Photochem. Photobiol.*, **45**, 587–594.